# Emerging properties of malaria transmission and persistence in urban Accra, Ghana: evidence from a participatory system approach

**DOI:** 10.1186/s12936-021-03851-7

**Published:** 2021-07-19

**Authors:** Merveille Koissi Savi, Daniel Callo-Concha, Henri E. Z. Tonnang, Christian Borgemeister

**Affiliations:** 1 Center for Development Research (ZEF), University of Bonn, 53113 Bonn, Germany; 2 Institute for Environmental Sciences (iES), University of Koblenz-Landau, 76829 Landau, Germany; 3 International Centre for Insect Physiology and Ecology (Icipe), Nairobi, Kenya

**Keywords:** Network analysis, Causal loop diagram, Emergence, Urban, Complex system

## Abstract

**Background:**

Several studies that aim to enhance the understanding of malaria transmission and persistence in urban settings failed to address its underlining complexity. This study aims at doing that by applying qualitative and participatory-based system analysis and mapping to elicit the system’s emergent properties.

**Methods:**

In two experts’ workshops, the system was sketched and refined. This system was represented through a causal loop diagram, where the identification of leverage points was done using network analysis.

**Results:**

45 determinants interplaying through 56 linkages, and three subsystems: urbanization-related transmission, infection-prone behaviour and healthcare efficiency, and *Plasmodium* resistance were identified. Apart from the number of breeding sites and malaria-positive cases, other determinants such as drug prescription and the awareness of householders were identified by the network analysis as leverage points and emergent properties of the system of transmission and persistence of malaria.

**Conclusion:**

Based on the findings, the ongoing efforts to control malaria, such as the use of insecticide-treated bed nets and larvicide applications should continue, and new ones focusing on the public awareness and malaria literacy of city dwellers should be included. The participatory approach strengthened the legitimacy of the recommendations and the co-learning of participants.

**Supplementary Information:**

The online version contains supplementary material available at 10.1186/s12936-021-03851-7.

## Background

Malaria is still the deadliest infectious disease, responsible for more than 380,000 deaths in 2018 only [1]. In endemic areas of sub-Saharan Africa (SSA), the expenses for its control and prevention reach up to 40% of all public health expenditures [2], and its effects were estimated to have reduced the GDP by 9% in 2010 in affected countries in SSA [3]. Due to the collaborative efforts of governments and development partners, malaria mortality has been reduced by 66% from 2007 to 2017 [4], but the challenge is yet far from being solved.

Africa’s population is expected to triple by 2050 [5], with major growth occurring in urban areas. For example, the population of major Ghanaian cities has grown by 3.5% per annum from 1984 to 2010 [6]. Urbanization has shifted the priorities of the public health system from the control of vector-borne diseases such as malaria to environmental public health challenges, such as traffic congestion, slumming, and pollution [7]. For example, in Accra, the capital of Ghana, urbanization-related issues often overshadow the infectious diseases related ones, with the local government investing less than 50USD per person per year on health [8]. Moreover, the poorest communities experience the greatest harm [8], like Accra’s head porters, especially challenged by the nature of their work and often not able to afford the national insurance scheme [9].

A pressing public health issue is the prevalence of vector-borne diseases, like malaria [10, 11]. Its predominant vector *Anopheles gambiae*, whose customary habitat for reproduction used to be rural, clean, and shallow water ponds surrounded by grassy fields, has now adapted to the urban conditions and prospers in polluted waters, such as clogged gutters or puddles, characteristic of poor urban housing [10, 12].

This example shows the complex and adaptive character of the malaria transmission system, where the humans, vectors, the environment, and parasites interact in an iterative and nonlinear manner [13]. Earlier modelling approaches rarely considered such complexity, and instead conceived transmission causal and unilaterally, which contributed to the development of policies favoring the promotion of single-intervention programmes, like the free provision of malaria drugs [14–16].

A complex system is one where its components, apparently disconnected and performing their roles after their interests, align together to perform more sophisticated functions [17]. This tends to be the case for most social and ecological phenomena, as they do not occur in isolation but intermingled [18]. The unraveling of complex systems is operationalized through approaches, methods, and tools that instead of assessing the determinants individually, focus on the interactions among them, and the overall function of the system [19]. In that regard, the involvement of local stakeholders appears key, as it reduces the bias of researchers and increases the legitimacy of the outcomes [20].

In this study, the interactions among the determinants of malaria transmission in urban conditions were visualized using a Causal loop diagram (CLD) and emergent properties of the system were displayed via Network Analysis (NA).

CLD can support the visualization of the interplay among determinants and assist in the identification of causal relationships among them [21]. Moreover, to better understand the complexity of malaria transmission, CLD can also help to devise improved strategies for more effective control of the disease in cities (and beyond) by scouting underplayed channels [13].

NA is based on the principles of network theory, where a system is considered a web of edges (interactions) that connect the nodes (determinants) [22]. The examination of the network properties (e.g. density) and its topology, e.g. centrality, indicate the nature of the information flow and reveal the most sensitive determinants and their roles [23, 24]. Furthermore, by applying graph theory, NA offers powerful visualizations of the analysed phenomena [22, 25]. A successful application of NA can display some emerging properties of complex systems, such as McGlashan [24] did in identifying the leverage points, i.e., where one can intervene to alter the system of childhood obesity in Australia.

It can be hypothesized that by combining participatory CLD and NA, the aims of this study, that are (i) to understand the interplay between determinants of the system of transmission and persistence of malaria in urban settings, and subsequently (ii) to identify its emerging properties (i.e., properties of the network and leverage points of the system and derive potential interventions on the system) would be accomplished.

## Methods

### Study area

Accra, the capital city of Ghana, is located on the coast of the West African Gulf of Guinea. Its climate is tropical alternating wet and dry phases, mainly due to the cyclical harmattan winds. The average annual rainfall is 730 mm and bimodally distributed, the temperature average reaches 26.6 °C, and the relative humidity rounds 81%, with little variations along the year [26, 27]. Accra’s current population is 2.3 million, to a great extent composed of migrants from successive waves of rural–urban migration across the last 50 years. Housing is uneven in infrastructure quality and service provision, but standards are generally low. The worst affected areas are old central neighborhoods, where slums abound, and peripheral settlements, where new developments happen [28].

Although malaria is traditionally considered a predominantly country-side disease, recent evidence showed that mortality and morbidity in SSA’s urban and rural areas are highly heterogeneous [5, 29, 30]. For example, in Accra, slums and poorly-managed urban areas such as James-Town and Korle-Dudor districts recorded the highest malaria indices of morbidity and mortality [31].

### Identification of key experts and causal loop diagram elicitation

Initially, an informal meeting was held with district assembly members of James-Town and Korle-Dudor districts and other members of the communities, to identify the key institutions and experts working on the prevention and treatment of malaria. The list of institutions and experts was consolidated to include twelve representatives from the Ghana National Malaria Programme, Malaria Initiative/USAID, World Health Organization, Ghana Health Service, Plant Protection and Regulatory Services/Ministry of Food and Agriculture, Noguchi Memorial Institute for Medical Research, several NGOs, and local healthcare facilities (Additional file 1: Table S1).

The experts met in two recorded qualitative workshops, which were facilitated by a modelling team (modeller, facilitator, and wall-builder) following Hovmand’s guidelines [32]. The architecture of the workshop is documented by [32, 33]. Most specifically, in the first workshop, the problem was refined, defined the variables of the model using five thematic clusters (vector, parasite, environment, human, and health care system), and drew an initial CLD. A CLD aims to show the interplay between components of a complex system, eliciting the feedback loops, and facilitate the understanding of a given problem [34, 35]. For that, the background was set by presenting the outcomes of precedent informal interviews; then, together with the experts, the boundaries of the malaria-related transmission and persistence CLD were defined. A time horizon of ten years was set to guide the discussion and the modelling. Consequently, determinants that are not very specific and have long time effects on the overall system (e.g., climate change) were removed from the discussion. However, their specific parameters (e.g. rainfall and temperature) were included.

**Fig. 1:**
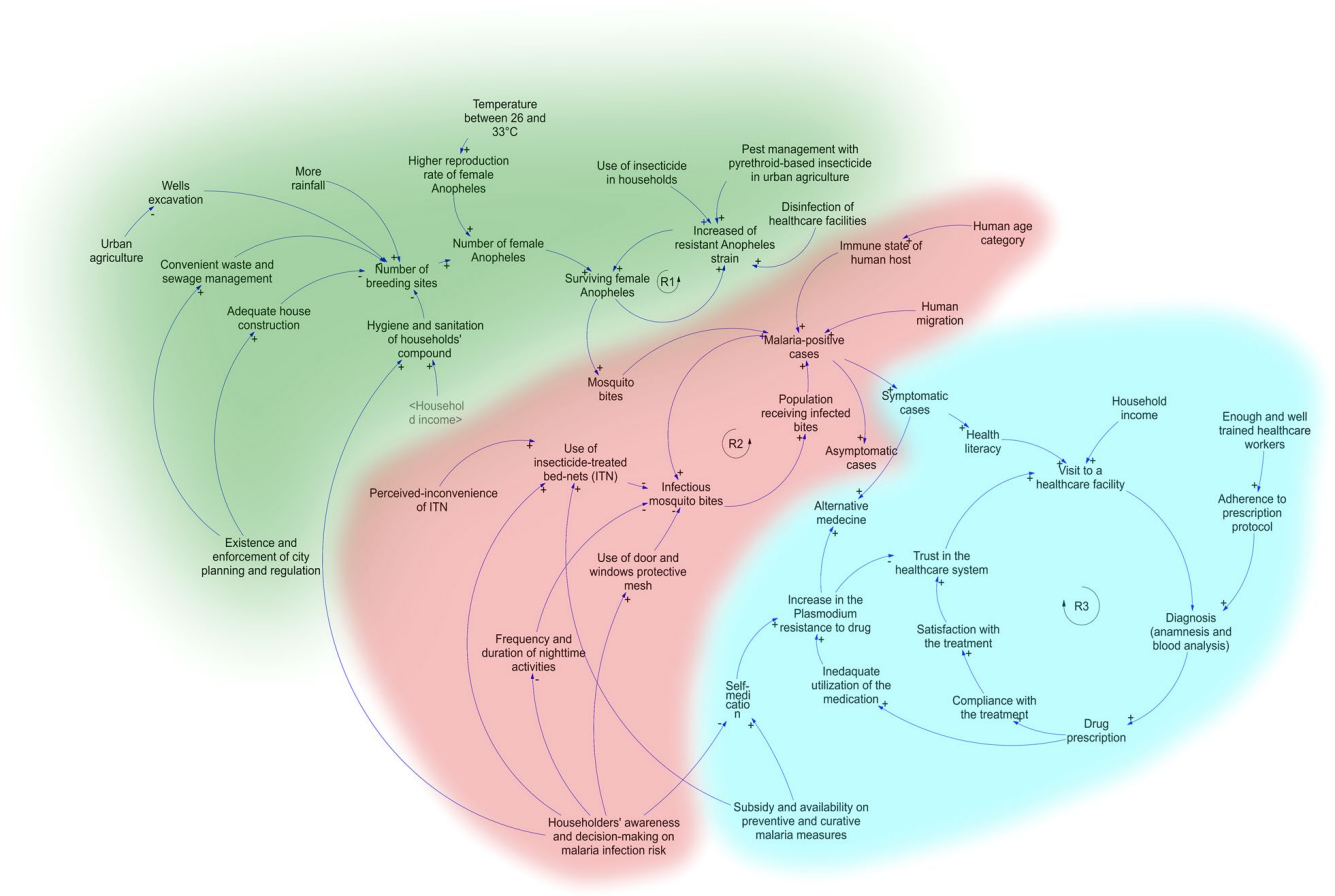
Causal loop diagram depicting the complexity in malaria transmission and persistence in Accra (Ghana). In green the urbanizationrelated transmission sub-model, in red the human's infection-prone behavior of malaria sub-model, and the healthcare efficiency and Plasmodium resistance sub-model (blue)

In the CLD, a *cause* is a determinant from which the arrow emerges, and an *effect* a determinant that receives the arrow. The positive or negative sign of the arrow explains the type of association, i.e., a cause A implying an effect B showing a positive sign should be read: An increase in A implies an increase in B. Inversely, A implying B with a negative sign should be read: an increase in A causes a decrease in B (Fig. 1). Subsequently, some determinants that were not locally relevant, e.g., indoor residual spray (not used in Accra) were excluded and exogenous determinants were limited to the minimum as suggested [36].

During the second participatory workshop, the CLD was refined and validated. Thus, new determinants were added whereas others were merged into more inclusive ones, and some determinants judged non-relevant were removed, which led to changes in the causal linkages. The model obtained (Additional file 1: Fig. S1) was fine-tuned by the modelling team based on the recordings. All expert participants were informed and agreed to be recorded and consented to the scientific use of those recordings.

### Network analysis

The emerging properties of the system represented by the CLD were displayed by the properties of the network and its most central determinants. The most central determinants stand for the leverage points for transmission and persistence of malaria in Accra. These points can enhance the control of malaria when they are adjusted according to the properties displayed by the system.

The CLD represents an unweighted directed network $G = \left( {V,E} \right)$, where $V$ and $E$ are respectively the set of the nodes and the edges. The connectivity in $G$ is represented by the adjacency non-symmetric and unweighted matrix $A_{ij}$ (Eq. 1) [24],

1 $$A_{ij} = \left\{ {\begin{array}{*{20}l} {1, if \left\{ {i,j} \right\} \in E} \\ {0, otherwise} \\ \end{array} } \right.$$

The properties of the network were estimated through the computation of the density, the average path length, and the modularity of the CLD network. The functional importance of the determinants was captured via the calculation of six measures of centrality, i.e., degree ($$K)$$, in-degree $$(K^{in} )$$, out-degree $$(K^{out} )$$, PageRank $$\left( x \right)$$, closeness $$\left( C \right)$$, and betweenness $$\left( B \right)$$.

The degree centrality ($$K)$$ assesses the determinants' connectivity. With respect to the adjacency matrix, the degree $$K_{i}$$ can be calculated for a network $$G$$ containing $$N$$ nodes:

2 $$K_{i} = K_{i}^{in} + K_{j}^{out}$$

where

3 $$K_{i}^{in} = \mathop \sum \limits_{j = 1}^{N} A_{ij} , K_{j}^{out} = \mathop \sum \limits_{i = 1}^{N} A_{ij} ,$$

$K_{i}^{in}$ and $K_{j}^{out}$ stand for in-degree and out-degree centralities and indicates the direction of the connection between determinants, the former as a recipient (effect), and the latter as emitter (cause).

PageRank centrality $x_{i}$ estimates the influence of certain determinants on the whole network [37, 38] and is given by

4 $$x_{i} = 0.85 \times \mathop \sum \limits_{j} A_{ij} \frac{{b_{j} }}{{k_{j}^{out} }}$$

where $x_{i}$ is the out-degree of the node $i$. Thus, $b_{j}$ is given by $$b_{j} = \left\{ {\begin{array}{*{20}l} 0 \\ 1 \\ \end{array} } \right.\begin{array}{*{20}c} {\quad if\; in - degree} \\ {otherwise} \\ \end{array}$$.

The closeness centrality $C_{i}$ calculates the proximity among determinants and identifies which one spreads more efficiently information in the network [23, 38–40]. $C_{i}$ is defined by

5 $$C_{i} = \frac{n}{{\mathop \sum \nolimits_{j} g_{ij} }}$$

where $n$ represents the total number of the shortest paths (the shortest self-avoiding route that runs from one determinant to another along with the connectivity [38]) between the determinant $i$ and $j$, and $g_{ij}$ is each elementary shortest path or the distance between the determinants $i$ and $j$.

The betweenness centrality $B_{i}$ measures how a determinant serve as a bridge between different part of the network [24, 37, 38, 41]. Assuming that $g_{st}$ is the total number of shortest paths from $s$ to $t$ then $n_{st}^{i}$ is the number of shortest paths from the determinants $s$ to $t$. Simply.

$$n_{st}^{i} = \left\{ {\begin{array}{*{20}l} {1 } \\ 0 \\ \end{array} {\kern 1pt} \quad \begin{array}{*{20}l} {if\; there\; is\; a \;relationship\; between \;s \;and \;t} \\ {otherwise} \\ \end{array} } \right.\quad$$.

The computation of $B_{i}$ is given by

6 $$B_{i} = \mathop \sum \limits_{st} \frac{{n_{st}^{i} }}{{g_{st} }}$$

All the analyses were run using R [42].

All participants involved in this study were informed and signed their consent for the recording and the use of the meetings’ materials for scientific purposes.

## Results

### A system model of malaria transmission and persistence

The transmission and persistence of malaria in Accra are portrayed in a CLD of the complex system model, entailing 56 interactions among 45 determinants (Additional file 1: Table S1). This model shows three sub-models triggered each by a reinforcing loop, i.e., (i) the urbanization-related transmission and acquired resistance of *Anopheles* to insecticides (green), (ii) the human's infection-prone behaviour (red), and (iii) the healthcare efficiency and *Plasmodium* resistance (blue) (Fig. 1).

#### Urbanization-related transmission and resistance of Anopheles to insecticides

The deficient city planning and planning enforcement, inadequate housing conditions, and limited waste and sewage infrastructure lead to the proliferation of *Anopheles* breeding sites, which is worsened by the excavation of wells for urban and peri-urban agriculture and rainfall. Besides, a temperature range between 26 and 33 °C in Accra, contributes to the increase in the reproductive rate of *Anopheles*, their absolute numbers, and finally their survival, augmenting the risk of infection.

Furthermore, the preventive use of insecticides in households, agricultural sites, and healthcare facilities leaves residues that contribute to the development of insecticide resistance in local mosquito populations. Thus, in the reinforcing loop one (R1, Fig. 1), the transmission of malaria depends not only on the environmental factors, such as temperature and rainfall but also on the lack of regulations to prevent and control the proliferation of mosquito breeding sites. These effects are exacerbated by the widespread use of insecticides. Hence, this reinforcing loop portrayed the environment as a pathway of both, the infection and the development of resistance of mosquitoes to insecticides (Fig. 1).

#### Humans infection-prone behaviour

At the individual and household levels, ideally, the awareness of malaria risk leads to the reduction of nighttime activities, as well as the use of protective/preventive measures against mosquito bites, such as the use of insect-proof mesh for doors and windows and insecticide-treated bed-nets (ITNs). The more these measures are accepted and used, the lower the infection will be. In addition, human migration increases the number of infected cases by importation. More infected people in Accra imply a greater number of mosquitoes becoming infected that will subsequently transmit the pathogens to new hosts. This reinforcing loop two (R2, Fig. 1) highlights the importance of individual and household decisions, and how a changing behaviour can prevent the transmission and its persistence by, for instance, reducing the nighttime activities and using protective measures like ITNs. (Fig. 1).

#### Healthcare efficiency and *Plasmodium* resistance

Malaria carriers can be asymptomatic, and as Ghana’s healthcare system is often unable to detect them, they frequently remain untreated and thus keep spreading the disease. Knowledge of malaria symptomatology and a sufficient household income lead to more visits to healthcare facilities. If healthcare workers are well trained, adhere to prescription protocols, and patients comply with the prescribed treatment, the reinforcing loop three (R3, Fig. 1) will operate, and trust in the health system will grow. If patients do not trust the health system, the use of inadequate medication and misuse of adequate medication will increase, and alongside the resistance of the *Plasmodium* parasite to preventive and curative drugs. Besides, the free availability of heavily subsidized drug treatments augments self-medication and indirectly enhances drug-resistance development in *Plasmodium*. This loop reveals that a good healthcare system requires to be well endowed logistically and in terms of personnel. Substituting such a healthcare system with highly accessible low-priced drugs can increase the prevalence of resistant strains of *Plasmodium*. Moreover, this loop revealed an unintended pathway of malaria treatment policies [32], which, although well-intentioned, can be counter-productive. Relatedly, symptomatic patients, unsatisfied with allopathic treatments and drug resistance, may opt for alternative medicines, despite their often uncertain outcomes.

### Network analysis of the CLD

#### Properties of the network

The network representing the CLD displayed a structure of *small-world*, meaning that all determinants are not interconnected but are anyhow reachable by a small number of steps [43]. It shows an average path of 6.309, meaning that each determinant can reach any other on average through 6.309 paths. Still, it has a low density (0.028), presenting only 2.8% of possible edges in a completely interconnected network, and suggesting that a change in a determinant will have only a limited impact on the whole system. This indicates that despite its apparent complexity, there is a small connection path among determinants, allowing the information to spread rapidly [24, 44]. Furthermore, a modularity of 0.619 implies a structural clustering among determinants, indicating that acting on the determinants of the highest betweenness will have a spillover effect on the whole system [24]. In other words, an effective way to impulse a change in the system is to induce a change in the mediator.

#### Network metrics

The CLD has a *scale-free* distribution, meaning that its in-degree and out-degree metrics showed a heavy-tailed distribution with values ranging from 0 to 5 (Additional file 1: Fig. S2). Few nodes show 0 out-degree, indicating that most of the determinants influence other determinants. This configuration describes well real-world networks and suggests a high resilience of the system [45] (Table 1 & Additional file 1: Fig. S2). Thus, beyond the mediator of the system, the other leverage points also needed to be strategically adjusted to efficiently finetune the system.

**Table 1:** Metrics of the network analysis of determinants of transmission and persistence of malaria in Accra

Label	K	C	B	x	K^in^	K^out^
Existence and enforcement of city planning and regulation	2	0.116	0	0.004	0	2
Adequate housing construction	2	0.119	10	0.006	1	1
Convenient waste and sewage management	2	0.119	10	0.006	1	1
Urban agriculture	1	0.111	0	0.004	0	1
Wells excavation	2	0.119	20	0.008	1	1
**Number of breeding sites**	6	0.128	147	0.032	**5**	1
More rainfall	1	0.119	0	0.004	0	1
**Householders' awareness and decision-making on malaria infection risk**	5	0.266	0	0.004	0	**5**
Hygiene and sanitation of households' compound	3	0.119	16	0.007	2	1
Household income	2	0.200	0	0.004	0	2
Temperature between 26 and 33 °C	1	0.119	0	0.004	0	1
Higher reproduction rate of female Anopheles	2	0.128	19	0.008	1	1
Number of female Anopheles	3	0.140	192	0.039	2	1
Surviving of female Anopheles	4	0.153	262	0.080	2	2
Use of insecticide in household	1	0.140	0	0.004	0	1
Insecticide resistant Anopheles strain	5	0.134	34	0.050	4	1
Pest management with pyrethroid-based insecticide in urban agriculture	1	0.124	0	0.004	0	1
Disinfection of healthcare facilities	1	0.124	0	0.004	0	1
Mosquito bites	2	0.160	246	0.038	1	1
Use of insecticide-treated bed-nets (ITN)	4	0.160	23	0.011	3	1
Perceived-inconvenience of ITN	1	0.145	0	0.004	0	1
Frequency and duration of nighttime activities	2	0.160	2	0.005	1	1
Use of door and windows mesh	2	0.160	2	0.005	1	1
Infectious mosquito bites	5	0.177	313	0.047	4	1
Population receiving infected bites	2	0.209	298	0.044	1	1
**Malaria positive cases**	**7**	0.255	**328**	**0.086**	4	3
Human migration	1	0.214	0	0.004	0	1
Human age category	1	0.186	0	0.004	0	1
Immune state of human host	2	0.214	15	0.008	1	1
Asymptomatic cases	1	0.000	0	0.029	1	0
Symptomatic cases	3	0.250	273	0.029	1	2
Health literacy	2	0.250	224	0.017	1	1
Visit to a healthcare facility	4	0.296	255	0.057	3	1
Enough and well trained healthcare-workers	1	0.233	0	0.004	0	1
Adherence to prescription protocol	2	0.273	9	0.008	1	1
Diagnosis (anamnesis and blood analysis)	3	0.333	239	0.060	2	1
**Drug prescription**	3	**0.381**	205	0.056	1	2
Compliance with the treatment	2	0.222	61	0.028	1	1
Satisfaction with the treatment	2	0.242	27	0.028	1	1
Trust in the healthcare system	3	0.267	47	0.043	2	1
Inadequate utilization of the medication	2	0.267	68	0.028	1	1
Increase in Plasmodium resistance to drug	4	0.296	61	0.034	2	2
Alternative medicine	2	0.000	0	0.031	2	0
Subsidy and availability on the preventive and curative malaria measures	2	0.236	0	0.004	0	2
Self-medication	3	0.250	18	0.007	2	1

degree ($$K)$$, in-degree $${(K}^{in})$$, out-degree $${(K}^{out})$$, PageRank $$(x)$$, closeness $$(C)$$, and betweenness $$(B)$$; Values in bold represent the higher value of centrality

The network centrality metrics revealed that *malaria-positive cases*, was the determinant of higher centrality, either impacting or been impacted by seven determinants. Also, the *number of breeding sites* was impacted by five other determinants, ($k^{in} = 5$) namely: 1. more rainfall, 2. the hygiene and sanitation of householders’ compound, 3. the adequate housing construction, 4. a convenient waste and sewage management, and 5. the wells excavation. Conversely, the householders' awareness and decision-making on malaria infection risk was the main cause of transmission and persistence, as it impacts five other determinants ($k^{out}$ = 5) namely 1. the hygiene and sanitation of household compound, 2. the use of ITN, 3. the frequency and duration of nighttime activities, 4. the use of door and windows mesh, and 5. self-medication.

Predictably, the determinant of highest betweenness that connects most clusters of determinants is the *malaria positive cases* ($B$ = 328); and the one with the highest Page rank, and, thus, the most influential is the *malaria positive cases* ($x =$ 0.086). Also, the determinant with the greatest closeness centrality, i.e., with the shortest distance to all others is *drug prescription* $(C =$ 0.381) (Table 1).

Interestingly, determinants participating directly in the infection process, such as the *number of breeding sites,* the *malaria positive cases*, or not, such as the *drug prescription* and *householders' awareness and decision-making on malaria infection risk* are also important leverage points (influential points in the system where a small change in these determinants can induce a big change in the whole system [46]) that can affect the system.

## Discussion

### System model of malaria transmission and persistence in Accra

The most central determinants also standing for the leverage points of the system, aside from the environmental-related ones, are those resulting from the citizens’ *awareness of malaria infection risks*, and *household income,* and derived empowerment. These findings corroborate earlier studies that showed that poor-income households are more vulnerable to the disease, by facing a double burden: Malaria hotspots in Accra are in economically deprived communities, where malaria infection risk is additionally fueled by the economic needs, leading to community members to take up jobs that increase their exposure [47]; and, poor-income households spend relatively more of their earnings on the treatment of malaria than the higher-income ones [48].

This study also stresses the importance of an efficient healthcare system that is a structural issue in most countries of the Global South. The *trust in the healthcare system*, which can be reinforced through *training and supervision of health workers* on malaria diagnosis and treatment-related protocols, and the *adherence of the health workers* to it was also found particularly relevant. Supporting this, a system dynamics simulation on policies for improving neonatal health in Uganda demonstrated that the workload of healthcare workers affects the development of trust of patients, especially when it leads to long waiting times for attention [49]. Likewise, a study in England and Wales revealed that the reduction of waiting time built the trust of patients and enhanced healthcare efficiency [50]. On the other hand, the *non-adherence to the prescription and treatment protocols* by health workers can lead communities to underestimate malaria infection and increase its effects, which augments the distrust in the health system.

Relatedly, the *subsidy on anti-malarial drugs* instead of promoting a more holistic public health policy contributes to *patients’ self-medication* [51], highlighting the sometimes counterproductive effects of a sole focus of public health programmes on biomedical policies as this ignores the complexity of malaria transmission [52]. Previous research showed that patients' self-medication leads to arbitrary dosage and posology of anti-malarial drugs, and tends to exacerbate the symptoms and augments the morbidity and mortality of malaria [53]. Such situations are worse in poor-income households, where people often self-medicate with inadequate or counterfeit drugs, and/or inappropriate dosages and posologies at the onset of malaria symptoms [51].

Humans can be infected with *Plasmodium falciparum* and be symptomatic or asymptomatic. The latter is most often undiagnosed because random testing of the population is costly [54]. Besides, the conventional rapid diagnostic test is not sensitive enough to allow efficient screening of low-level parasitemia observed in asymptomatic infections [55]. This contributes to feeding a permanent human reservoir of *P. falciparum* and thereby contributing to the persistence of malaria [56]. Also, imported cases by human migration amplify the number of cases in cities and at times can initiate the resurgence of malaria in locations where it had been previously under control [57, 58].

These findings also suggest that deficient urban sanitation and poor urban planning increase the number of mosquito breeding sites. About 10% of *Anopheles* mosquito breeding sites in Accra are situated around construction sites [12]. Similar observations were made in Nigeria and Tanzania, where clogged gutters and sewage channels are playing similar roles [59]. Thus, the ecology- and behaviour-related adaptations of the mosquitoes delay control and make such efforts less effective, thereby contributing to the persistence of malaria in cities [60].

### Emergent properties of malaria transmission

The *small-world* and *scale-free* properties that feature the CLD indicate that the network is resilient and the identified leverage points could help to set more adequate policy recommendations.

The functional analysis of the network allowed to identify the determinants of the more central standing for potential intervention points such as causal, impacted, spreader, mediator, and influential, and derive from them key leverage points. Thus, the determinant (i) the *malaria-positive cases* was both, the most influential and the greatest mediator; and (ii) the *number of breeding sites* had the larger effects. These findings align with a recent review of malaria determinants for sub-Saharan Africa [61] that highlights the surge of infection as an intricate interplay between mosquitoes, humans, and their environments. Furthermore, (i) the *drug prescription* was the determinant with the highest closeness centrality, and (ii) the *householders' awareness and decision-making on malaria infection risk* the most important cause. This indicates that malaria transmission and persistence rely heavily on human behaviour, which opens opportunities for more targeted policy action.

The CLD was able to disclose the interactions among malaria determinants and also permitted to track the causal links among them that preserve transmission and the feedback loops that reinforce certain sets of determinants [62], which permits signaling emerging properties of the system post the NA [63].

## Limitations of the study

The CLD is a systemic tool capable to depict how determinants are interconnected. In this case study, the CLD has been collectively built enabling the generation of a group thinking model [35]. As such, CLD may preclude its replicability and reproducibility because the model is contingent on the experts’ perception of the system. Therefore, for the same problem, more determinants can be always identified. Thus, the CLD is an over-simplification of the real-world system. Nonetheless, Richardson [64] argues that CLD still contains information that could be further transferred to the decision-makers as it is meant to embody the premises underpinning the functioning of the system [65].

On the other hand, CLD is described as a qualitative and often perception-based model that should be read as a causal-effect model [36]. As such it is based on a set of qualitative interviews, workshops, or qualitative reviews. For instance, it has been used to document the interplay between factors that lead to childhood obesity in the US [66], to display the obesity-related behaviour in youth [67], and to highlight a pathway for the prevention and the response to covid-19 [68]. Since the CLD is not sustained by empirical observations [36], it leaves room to question the causal inferences drawn from its interpretation. Nonetheless, this study shed light on potential avenues for forthcoming empirical test the causality among the determinants of the transmission and persistence of malaria in Accra and other urban settings.

The validation of the topology of a network is often carried out through structural modifications of the network using random addition and removal of nodes and edges [69–71]. This operation allows displaying the resilience of the network [71]. It has been extensively used in social networks and protein network analyses to validate the most important nodes (after re-computation of the centrality metrics) without ruining the underlining problem of friendship building or elaboration of proteins. However, as the CLD is a thematic network, a structural modification of the network will also lead to a functional change of the network deviating from the identification of the leverage point of a system of transmission and persistence of malaria in Accra. Nonetheless, the calculation of the metrics combined with the properties of the network enables the identification of potential strategies that may guide policy recommendations for better control of malaria.

## Conclusions

The proposed CLD contributed to illustrate the complexity of malaria transmission and persistence in this study, Accra, Ghana. It showed that beyond the mere biological processes and the physical environment, the behaviour of people plays a key role in malaria transmission and persistence. The CLD embodies three major loops that trigger and maintain transmissions in urban environments. Furthermore, the NA enabled the detection of emergent properties of the system and the identification of the key levering determinants. Besides, the topology disclosed by the CLD revealed that all leverage points need to be accounted for strategic policy development. Hence, major efforts toward preventing malaria transmission are needed, and on that, the key priorities should be: to reduce malaria persistence by reducing mosquito density, for instance, through the regular drainage of gutters or treating breeding sites with larvicides; and reducing infections by increasing the awareness of city dwellers on malaria literacy, for instance, through regular campaigns in deprived communities, both, on the field and social media. Ongoing measures, like, protecting windows and doors with mosquito-proof netting and the use of ITNs, should be intensified. Besides, an improvement of the healthcare system through regular training of the healthcare workers in malaria can enhance trust in the healthcare system and limit the risk of patients' non-compliance to malaria-drugs prescription.

## Supplementary Information

**Additional file 1.** Additional Tables and Figures.

## Data Availability

All relevant data are provided in the manuscript or available from published materials as cited.

## Abbreviations

CLD: Causal loop diagram
ITNs: Insecticide-treated bed-nets
NA: Network analysis
NGO: Non-governmental organizations
SSA: Sub-Saharan Africa
USAID: United States Agency for International Development
