# Supplementary material for: Emerging properties of malaria transmission and persistence in urban Accra, Ghana: evidence from a participatory system approach
Source: Malar J. 2021 Jul 19;20:321. doi: 10.1186/s12936-021-03851-7 (PMC8287558; doi:10.1186/s12936-021-03851-7)
Supplement: Supplementary file 1 — Additional file 1. Additional Tables and Figures. [file 12936_2021_3851_MOESM1_ESM.docx]

Emerging properties of malaria transmission and persistence in urban Accra, Ghana: Evidence from a participatory system approach

Merveille Koissi Savi ^1, *^, Daniel Callo-Concha ^1,2^, Henri E.Z. Tonnang ^3^ & Christian Borgemeister ^1^

Additional file

Table S1: Composition of the group building sessions

| Institutions | Field of expertise | Number of participants |
| --- | --- | --- |
| Ghana National Malaria Program | Entomology surveillance | 1 |
| Malaria Initiative/ USAID | Prevention campaign and ITN distribution | 1 |
| World Health Organization | Prevention and control | 1 |
| Ghana Health Service | Public awareness | 1 |
| Plant Protection and Regulatory Services/ Ministry of Food and Agriculture | Pest management | 1 |
| Noguchi Memorial Institute for Medical Research | Genetic | 1 |
| VectorWork | ITN distribution | 1 |
| Korle Bu Teaching Hospital | Physician | 1 |
| Dodowa Health Research Center | Research | 1 |
| School of Public Health Ghana | Medical geography and malaria expert | 1 |
| Greater Accra Municipal Assemblies | Community service | 1 |
| Ghana National Malaria Program | Prevention and control | 1 |

Table S2: Operationalization of the determinants

|  | Label | Operationalization |
| --- | --- | --- |
| 1 | Existence and enforcement of city planning and regulation | Legal dispositions regulating the construction and renovation of buildings in cities. e.g., laws preventing mosquito breeding sites in building/renovation activities. It also includes disposition on urban planning. |
| 2 | Adequate housing construction | House building after the recommendations of the city planning offices |
| 3 | Convenient waste and sewage management | Regular waste collection and disposal, and existence and maintenance of drain and gutters for sewage evacuation |
| 4 | Urban agriculture | Farming activities carried out nearby urban housings. Usually associated with the use of pesticides, digging of wells, and also hosting conditions |
| 5 | Wells excavation | For irrigation purposes, which also offer sites for mosquito egg-laying |
| 6 | Number of breeding sites | Temporary or permanent pits and ponds that can offer breeding conditions for mosquitoes |
| 7 | More rainfall | Between 800-1200 millimeters a year, optimal for mosquitoes mating and reproduction |
| 8 | Householders' awareness and decision-making on malaria infection risk | People information and empowerment of measures leading to prevent the infection and properly treat malaria |
| 9 | Hygiene and sanitation of households' compound | To preventing mosquito breeding sites |
| 10 | Household income | Financial/monetary income of the household, correlated to higher capabilities to acquire medicines, look for medical treatment, etc. |
| 11 | Temperature between 26 and 33°C | Ideal temperature or mating and the eggs-laying of mosquitoes |
| 12 | Higher reproduction rate of female Anopheles | As female mosquitoes are the ones that transmit malaria, their relative increase relates to mating, egg-laying, and blood-sucking activities. A mosquito carried the parasites bite a human and inject into him the parasites. Through multiplication firstly in the leaver and then in the bloodstream, the parasite creates a series of symptoms. These symptoms can be expressed as fever, chill, sweating in that the person is symptomatic. In some cases, the person carrying alongside the parasite may not express any symptom and this person is denoted as asymptomatic. |
| 13 | Number of female Anopheles | Related to 12 |
| 14 | Surviving of female Anopheles | Related to 12 |
| 15 | Use of insecticide in household | Specific insecticide is used in the household to kill mosquitoes, which in the long-run these tend to increase their tolerance and eventually resistance |
| 16 | Insecticide resistant Anopheles strain | Eventually, mosquitoes mutate and get adapted to chemical compounds that are supposed to kill them |
| 17 | Pest management with pyrethroid-based insecticide in urban agriculture | Inadequate use and disposal of insecticide and pesticide-based pyrethroid pollutes the environment and drives the resistance of mosquitoes to insecticides. |
| 18 | Disinfection of healthcare facilities | Residuals of the pesticides applied remain in the environment and by their low doses augment mosquito resistance |
| 19 | Mosquito bites | Infection means, not all stings are infectious |
| 20 | Use of insecticide-treated bed-nets (ITN) | Used to repel mosquitoes and prevent infectious contacts with humans |
| 21 | Perceived-inconvenience of ITN | Acceptability of the ITN. Some populations allude as causes to reject them: increase in temperature, the itchiness of compound contains in the ITNs, the impossibility to breath, etc. |
| 22 | Frequency and duration of nighttime activities | Duration of the activities overnight can increase the exposure risk |
| 23 | Use of door and windows mesh | Indoor and windows to prevent mosquitoes entry |
| 24 | Infectious mosquito bites | Bite with a high likelihood of transmitting Plasmodium parasite to the human. Not all of the bites are infectious. |
| 25 | Population receiving infected bites | The fraction of the population that gets the infectious bite |
| 26 | Malaria positive cases | Population carrying Plasmodium parasite. This fraction of the population contains symptomatic and asymptomatic cases. |
| 27 | Human migration | Mobility of the population, which may imply the arrival of infected individuals |
| 28 | Human age category | The infectiousness is age-related, for example, children are more vulnerable to get infected than adults |
| 29 | Immune state of human host | Natural protection good health safeguard |
| 30 | Asymptomatic cases | The fraction of the population carrying the parasite but not expressing any symptoms of the disease |
| 31 | Symptomatic cases | Malaria clinical cases, so showing symptoms |
| 32 | Health literacy | Peoples level of information on how to prevent malaria infection and what to do in case been infected |
| 33 | Visit a healthcare facility | Check-up for diagnosis or treatment |
| 34 | Enough and well trained healthcare-workers | Personnel well-trained implies better and earlier malaria diagnostic and more timely and efficient treatment |
| 35 | Adherence to prescription protocol | The patient adherence to the prescribed treatments is related to a prompt and more comprehensive recovery |
| 36 | Diagnosis (anamnesis and blood analysis) | Proper and effective diagnosis, imply an adequate medicine prescription, often measures to optimize it: anamnesis and blood screening, are poorly implemented or absent |
| 37 | Drug prescription | Prescription of malaria drug by health personnel |
| 38 | Compliance with the treatment | Patient strict observance of the posology and prescribed doses of the prescribed medicines. Also, neglection is frequent |
| 39 | Satisfaction with the treatment | Patient recovery with the received treatment |
| 40 | Trust in the healthcare system | Reaction to the effectiveness of the healthcare system |
| 41 | Inadequate utilization of the medication | Misuse of drugs, which tends to weaken its efficiency |
| 42 | Increase in Plasmodium resistance to drug | By the inadequate treatment, non-compliance of treatment, etc. resistance of the parasite to the given medicine |
| 43 | Alternative medicine | All the alternative used to prevent and treat malaria, often traditional medicines like phytotherapy |
| 44 | Subsidy and availability on the preventive and curative malaria measures | There is an old tradition to subsidize malaria drugs and make them freely (no-prescription needed) available to the population |
| 45 | Self-medication | Use of medication without a formal prescription, quite strong in the case of anti-malaria drugs |


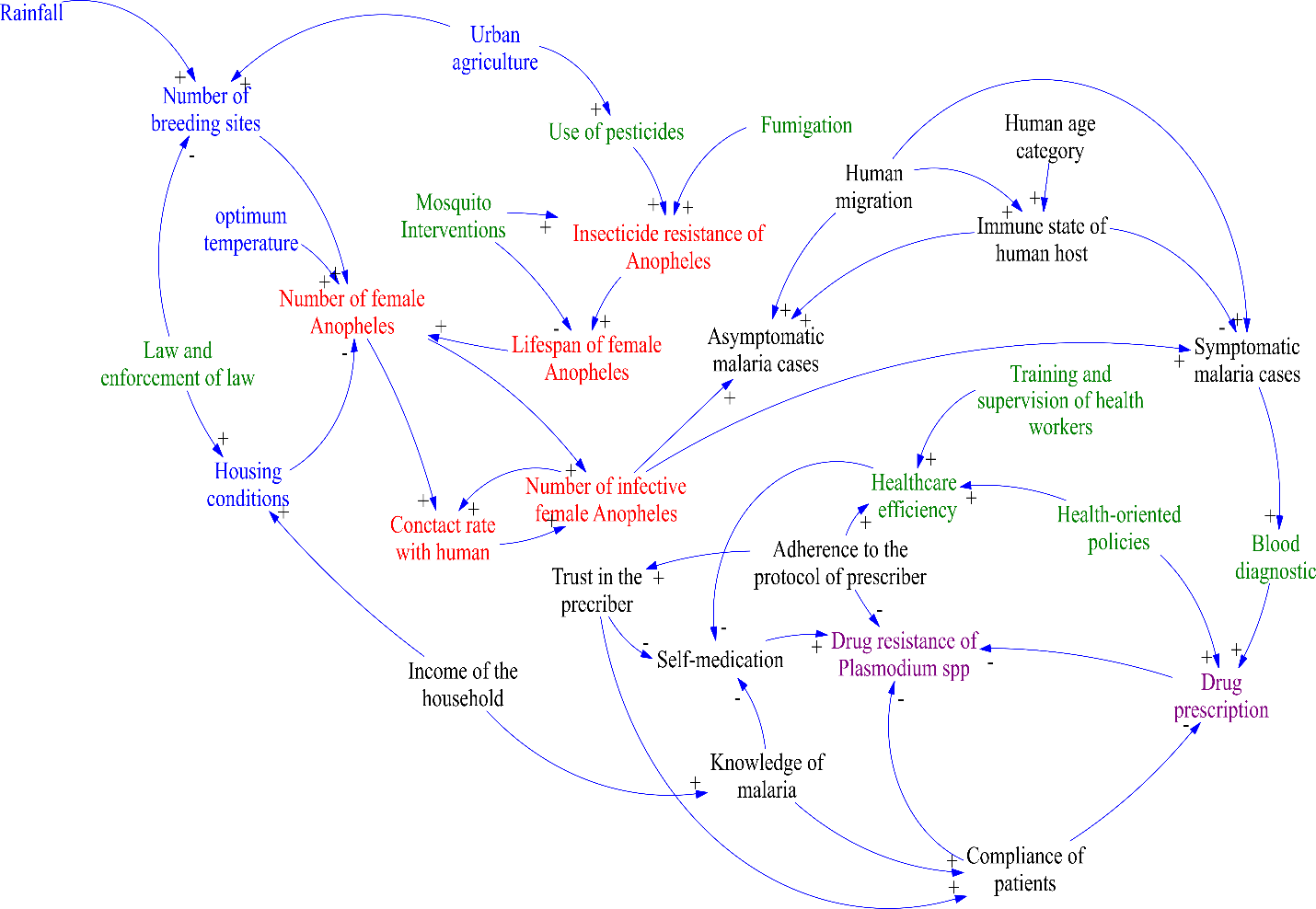


Fig. S1: Earlier system depicting the complexity of malaria in urban settings.

|  |  |
| --- | --- |

Fig. S2: In- and Out-degree distribution of node representing the complex system of malaria transmission and persistence in Accra, Ghana
